# Recognition of Dorsal Hand Vein Based Bit Planes and Block Mutual Information

**DOI:** 10.3390/s19173718

**Published:** 2019-08-28

**Authors:** Yiding Wang, Heng Cao, Xiaochen Jiang, Yuanyan Tang

**Affiliations:** 1 Department of Communication Engineering, School of Information, North China University of Technology, No. 5, Jinyuanzhuang Road, Shijingshan District, Beijing 100043, China; 2 Department of Computer and Information Science, School of Technology, University of Macau, Macau 999078, China

**Keywords:** bit planes, block, mutual information, cross-device, dorsal hand vein recognition

## Abstract

The dorsal hand vein images captured by cross-device may have great differences in brightness, displacement, rotation angle and size. These deviations must influence greatly the results of dorsal hand vein recognition. To solve these problems, the method of dorsal hand vein recognition was put forward based on bit plane and block mutual information in this paper. Firstly, the input gray image of dorsal hand vein was converted to eight-bit planes to overcome the interference of brightness inside the higher bit planes and the interference of noise inside the lower bit planes. Secondly, the texture of each bit plane of dorsal hand vein was described by a block method and the mutual information between blocks was calculated as texture features by three kinds of modes to solve the problem of rotation and size. Finally, the experiments cross-device were carried out. One device was used to be registered, the other was used to recognize. Compared with the SIFT (Scale-invariant feature transform, SIFT) algorithm, the new algorithm can increase the recognition rate of dorsal hand vein from 86.60% to 93.33%.

## 1. Introduction

Biometric is a technique that uses inherent and unique biometric feature to recognize people identification [1]. Biometric authentication systems are well established today as they exhibit many advantages over traditional password and token-based ones [2]. Dorsal hand vein recognition mainly uses the subcutaneous vein tissue structure of the dorsal hand for personal identification, the vein structure of the back of hands is highlighted because of the different infrared light absorption rates [3]. Anatomical works [4] have proved that the structure of dorsal hand vein is unique in the process of growth and development. Therefore, research on the recognition of the dorsal hand vein is becoming more and more important in terms of value.

In recent years, more and more researchers have begun to pay attention to the algorithm of hand vein recognition. These algorithms for feature extraction are roughly divided into global and local texture features. The global texture feature, such as PCA(Principal components analysis, PCA) [5], it utilizes the geometric texture of the hand vein and the texture mapping of the ROI (region of interest), but to a certain extent, it ignores the local information which is separable. Its performance is easily affected by the change of viewing angle, illumination intensity, distortion and occlusion. Local texture feature, such as LBP(Local binary pattern, LBP) [6] and SIFT [7], pay attention to the relationship between key pixels and surrounding pixels, so the matching with local key features is more robust to the above-mentioned interference factors, Because the texture details of the hand vein is rather few, combining both a global and a local method was proposed and the performance has been improved. Zhang et al. proposed a Gaussian distribution based random key-point generation (GDRKG) [8] which can obtain a reasonable number of key points with good coverage, so it could improve recognition performance. Wang et al. proposed cross-device hand vein recognition based on improved SIFT [9] which is based on the traditional SIFT, but optimized for the scale factor σ, using an extreme searching neighborhood structure and matching threshold R. It not only has had a significant improvement in the recognition rate in single-device experiments, but also a higher recognition rate than the traditional SIFT in cross-device experiments. Li et al. proposed hand dorsal vein recognition by matching using a width skeleton model, which uses the width skeleton model (WSM) [10] containing width and structural information. It makes full use of the global shape information, making the ability to characterize vein features stronger.

Although the above methods have achieved a high recognition rate, research on the dorsal hand vein are mostly based on a database acquired by a single device. Considering the diversity of imaging acquisition devices, as well as changes in environment and growth, hand vein recognition is very limited. At present, most published research papers are carried out under the strong constraints of controlled environment and user cooperation to achieve higher recognition accuracy. How to improve the cross-device hand vein recognition rate in the condition of seldom cooperation for users is the main problem solved in this paper. We propose a feature extraction method based on bit planes and block mutual information. The optimal bit plane was selected to overcome the influence of brightness and noise. The texture features of dorsal hand vein were described by a block method, and the optimal number of blocks was determined by the average entropy matrix of different blocks. Then the mutual information among different blocks was calculated as texture features by three kinds of mutual information calculation modes. Finally, the Euclidean distance classifier was used for classification recognition. The recognition rate of dorsal hand vein images under a cross-device increased to 93.33%.

## 2. Bit Plane Generation

### 2.1. Image Acquisition of Dorsal Hand Vein

**Figure 1:**
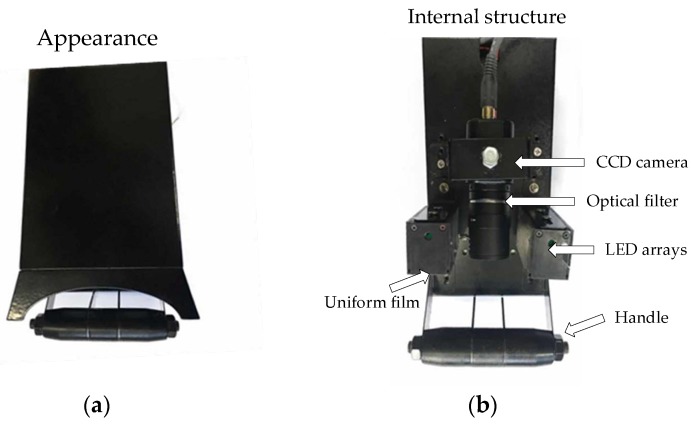
Image acquisition device. (**a**) Appearance; (**b**) internal structure.

**Figure 2:**
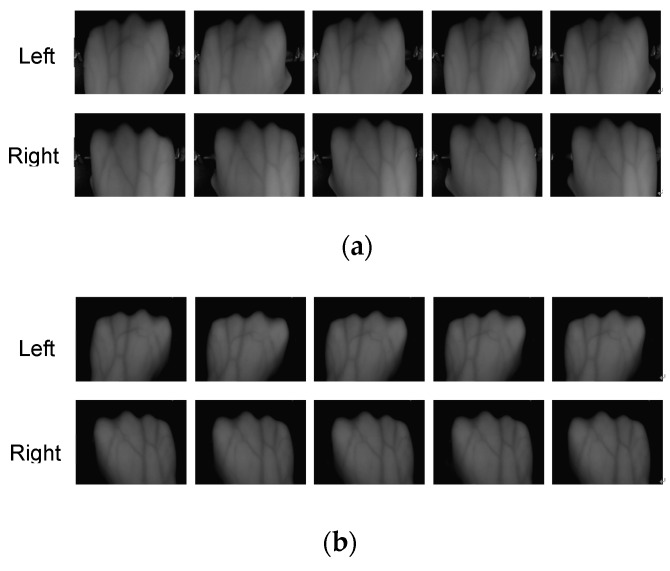
Dorsal hand vein images captured by two different devices. (**a**) Captured by No. 1 device; (**b**) captured by No. 2 device.

The dorsal hand vein exists under subcutaneous tissues. Using a general camera is difficult to capture clear images of dorsal hand vein in the condition of visible light source, so an infrared light source was adopted in our devices [11]. The appearance of acquisition equipment is shown in Figure 1a,b is the internal structure. Figure 2 is the captured images, Figure 2a,b are both dorsal hand vein images for the same person but captured by two different devices.

As we can see in Figure 2, the vein texture is clear and the details are rich. In order to research the image recognition of the dorsal hand vein under weak constraints, we need to create a database with diversity. Dorsal hand vein images of the same person under different devices are quite different, including changes in rotation angle, size, brightness and noise. This is mainly due to differences in parameters such as contrast, brightness, focal length and lens optical performance of different collection devices, as well as in the state of the collector’s hand. Therefore, it is more difficult for cross-device dorsal hand vein recognition.

### 2.2. Image Preprocessing

**Figure 3:**
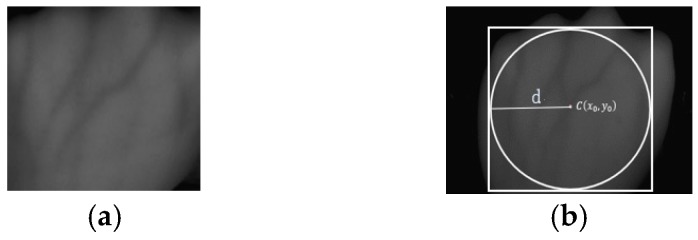
Captures the region of interest (ROI) area. (**a**) Maximum inscribed circle; (**b**) ROI area.

As mentioned above, dorsal hand vein images captured by the same person on different devices also have great differences in brightness, noise, size and rotation angle [12]. These factors will have a great impact on the recognition results, and simple scale normalization is not conducive to extract texture features of samples. In this paper, a centroid adaptive method was used to determine the ROI region of dorsal hand vein images. Find the centroid $C\left(x_{0},y_{0}\right)$ of the ROI hand vein image through the length and width of the vein area, and the centroid was taken as the center of the maximum inscribed circle of dorsal hand vein area which is shown in Figure 3a. The diameter (d) of the maximum inscribed circle, was taken as the standard of size normalization. After scale normalization, the ROI region with a size of 400 × 400 is intercepted, as shown in Figure 3b.

In addition, because of the difference about lighting condition and each thickness of hand, distribution of gray level in images can’t be equal. Thus, we need to normalize gray level from 0 to 255 by Formula (1).

**Figure 4:**
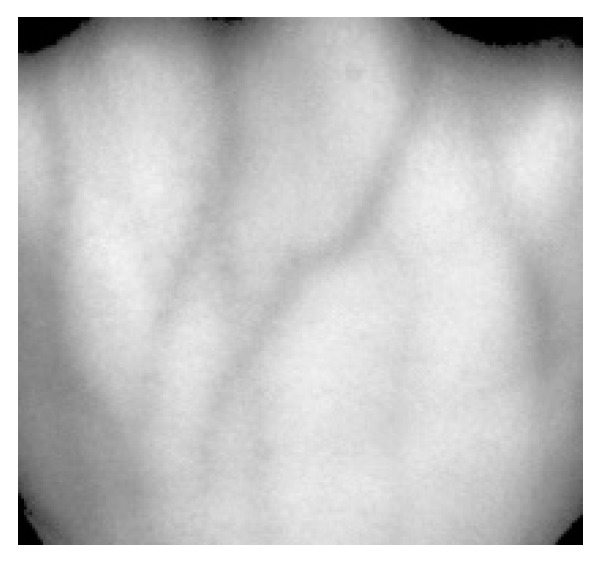
Gray-normalized ROI dorsal hand vein image.

(1) N(x,y)=((R(x,y)−min)×255)/(max− min)

where R(x,y) represents image gray level of the ROI region, max and min represent respectively the maximum and minimum gray value of images, N(x,y) represents the normalized gray level. The result after gray normalization is shown in Figure 4.

**Figure 5:**
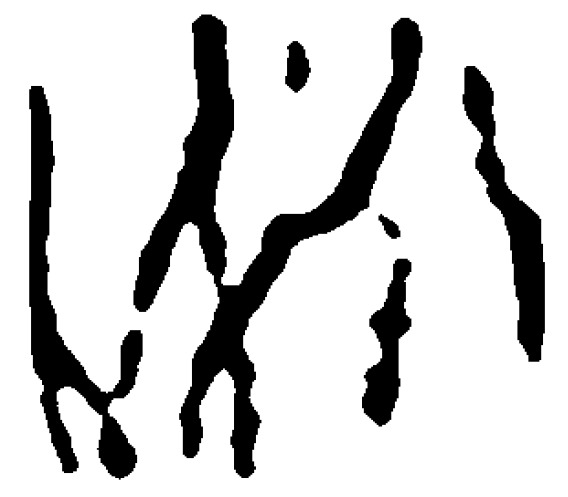
The segmented binary image.

In order to obtain the texture contour of dorsal hand vein, gradient based image segmentation method [13] was adopted in this paper. The segmented binary image is shown in Figure 5.

**Figure 6:**
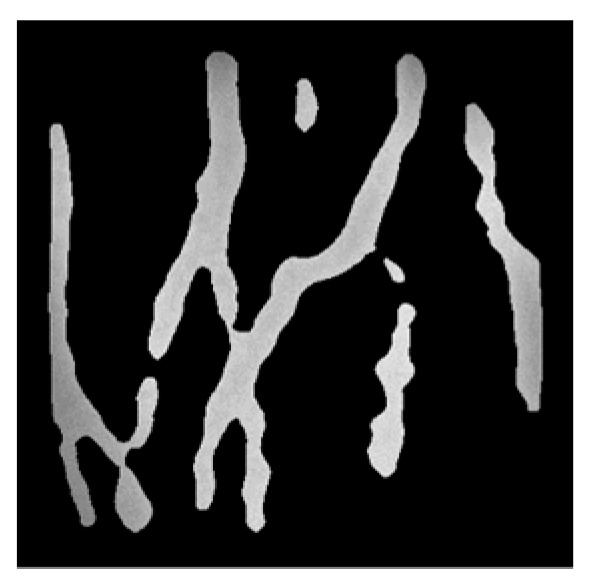
The gray image that only retains the contour of dorsal hand vein.

However, the binary image may lose lots of gray information, therefore, multiplying inverted binary image and normalized gray image to obtain the gray image that only retains the contour of dorsal hand vein, as shown in Figure 6.

### 2.3. Selection of Bit Plane

**Figure 7:**
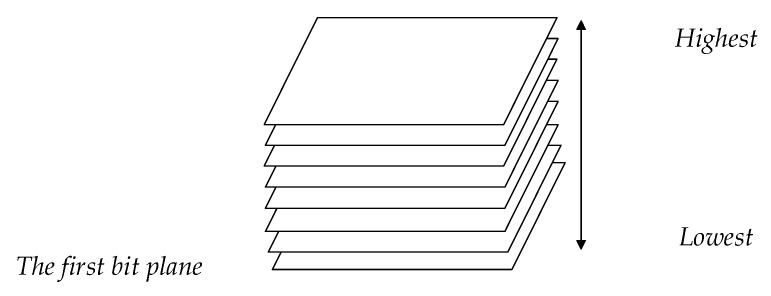
Bit plane stratification.

In order to obtain more abundant gray information and overcome the interference of brightness and noise caused by the collection environment, we studied the bit planes generated by gray image that only retains the contour of dorsal hand vein. The concept of bit planes is now illustrated by a 256-level gray image. If per pixel value of the input gray image is within [0, 255], then each pixel can be denoted by a binary number of eight bits, i.e., $b_{7},b_{6},b_{5},b_{4},b_{3},b_{2},b_{1},b_{0}$, as shown in Formula (2). From $b_{7}$ to $b_{0}$ are the highest to the lowest bit plane respectively as shown in Figure 7.

(2) $$I=b_{7}\times 2^{7}+b_{6}\times 2^{6}+b_{5}\times 2^{5}+b_{4}\times 2^{4}+b_{3}\times 2^{3}+\;b_{2}\times 2^{2}+b_{1}\times 2^{1}+b_{0}\times 2^{0}$$

**Figure 8:**
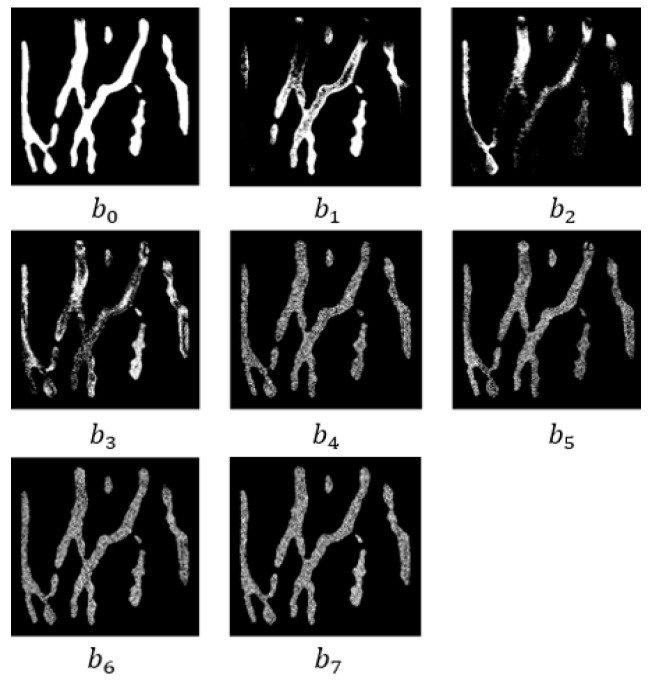
Eight-bit planes.

Each item in Formula (2) denotes a bit plane of a pixel, and eight bit planes are shown in Figure 8.

As we can see in Figure 8, the lower bit planes are close to binary images, which is easily interfered with by the noise from the collection environment and equipment, and the higher bit planes contain more gray information, which is close to the gray image that only retains the contour of the dorsal hand vein. It is susceptible to illumination and brightness during acquisition. Therefore, we chose the intermediate optimal bit plane to solve these problems effectively. In the following, the eight-bit planes are respectively divided into blocks to calculate mutual information, and the statistical recognition rate will be used to obtain the optimal bit plane to improve the accuracy and robustness of the hand vein recognition.

## 3. Hand Vein Recognition Based on Block Mutual Information

### 3.1. Mutual Information Calculation

Calculating the correlation of different bit planes and finding the best match is an important issue in this research. The correlation between different bit planes indicates the similarity of their contents, and their correlation can be characterized by mutual information [14].

For discrete random variables, let *X* be a random variable, *p*(*x*) is the probability that this variable *X* takes the value *x*, then the entropy H(*X*) describing its uncertainty is expressed as:

(3) $$H\left(X\right)=-\sum_{x\in X}p\left(x\right)log\;p\left(x\right)$$

The introduction of mutual information is to measure the amount of information that contains another random variable in a random variable, which denotes closeness between two random variables. With two random variables *X* and *Y*, the probability distributions are *p*(*x*) and *p*(*y*), respectively, and the mutual information between them is expressed as:

(4) $$I\left(X;Y\right)=-\underset{x\in X}{\sum }\underset{y\in Y}{\sum }p\left(x,y\right)\log\frac{p\left(x,y\right)}{p\left(x\right)p\left(y\right)}$$

Mutual information of images denotes the correlation between images [15], and it can be expressed as:

(5) $$I\left(A;B\right)=\underset{a\in K_{a}}{\sum }\underset{b\in K_{b}}{\sum }p\left(a,b\right)\log\frac{p\left(a,b\right)}{p\left(a\right)p\left(b\right)}$$

In Equation (5), *p*(*a*) and *p*(*b*) are respectively the probability distributions of image *A* and image *B*, *p*(*a*, *b*) is the joint distribution probability, $K_{a}$ and $K_{b}$ are gray levels. The larger I(A;B), the higher correlation between two images.

### 3.2. Optimal Number of Blocks

**Figure 9:**
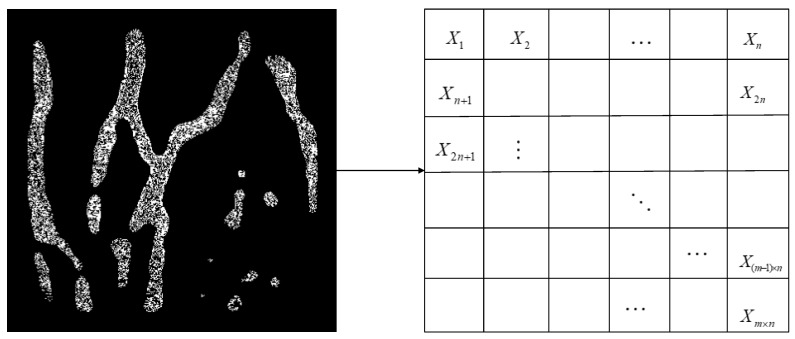
Divide image into blocks.

As mentioned above, the mutual information can indicate the correlation between images, however calculating that between each bit plane not only is a large amount of calculation, but also the information entropy obtained cannot distinguish different categories well. Therefore, we used a block method to describe the texture of dorsal hand vein, which not only solves the above problems, but in addition; the texture relationship between blocks can eliminate the effects of image rotation and scale changes. The image is divided into m × n blocks as shown in Figure 9.

The number of blocks will affect the extraction of texture features, the appropriate number of blocks can not only minimize dimension of the image, but also largely retain the texture information of the dorsal hand vein, so it is necessary to find the most appropriate number of blocks. According to the principle of pattern recognition, the optimal number of blocks should meet the requirement that the variance of the average entropy matrix based the average threshold as large as possible [16], so as to maximize the difference in average entropy between different blocks. In other words, the difference in texture information is obviously reflected and has good separability [17].

**Figure 10:**
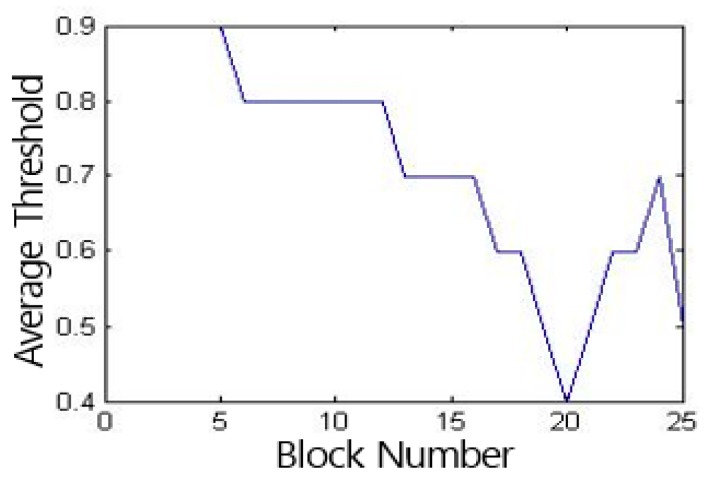
Average threshold distribution of average entropy matrix.

The image is divided from 1 × 1 to 25 × 25 blocks, and the grayscale symbiosis matrix of each sub-block is calculated to obtain the average entropy matrix of each image [18]. We used the Otsu method [19] to obtain the global threshold of each average entropy matrix, and then calculated the average threshold of all images under the same number of blocks, the result is shown in Figure 10.

As the number of blocks increases, the average threshold gradually decreases. This is because the sub-image becomes smaller as the number of blocks increases, so the energy of the grayscale symbiosis matrix is reduced. Calculate the corresponding variance according to the average threshold distribution of the average entropy matrix, the formula is:

(6) $$V=\sum {\left(f_{ij}-t\right)}^{2}$$

**Figure 11:**
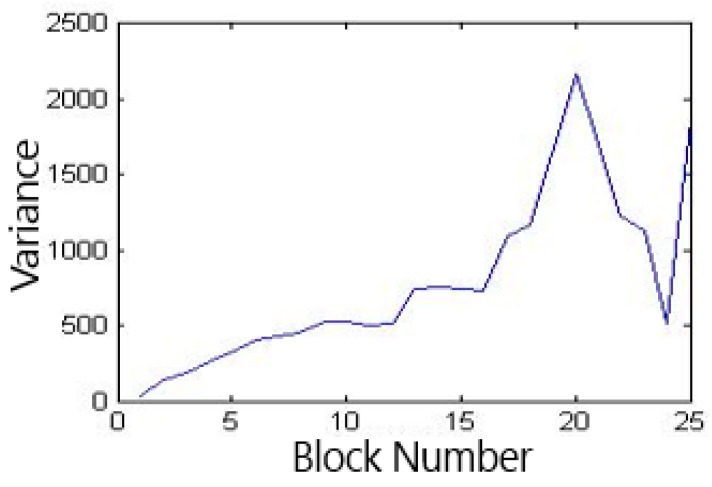
The variance of the average entropy matrix.

In Formula (6), t is the average threshold corresponding to average entropy matrix, $f_{ij}$ is the global threshold corresponding to average entropy matrix of each dorsal hand vein image, i is the category to which image belongs in this experiment, and j is the order in which image are arranged in this category, the result is shown in Figure 11.

It can be seen from Figure 11, that when the number of blocks is 20 × 20, the variance is the largest, that is, its threshold value is the best for the classification of the average entropy matrix.

### 3.3. Block-based Mutual Information Feature Vector Calculation Mode

**Figure 12:**
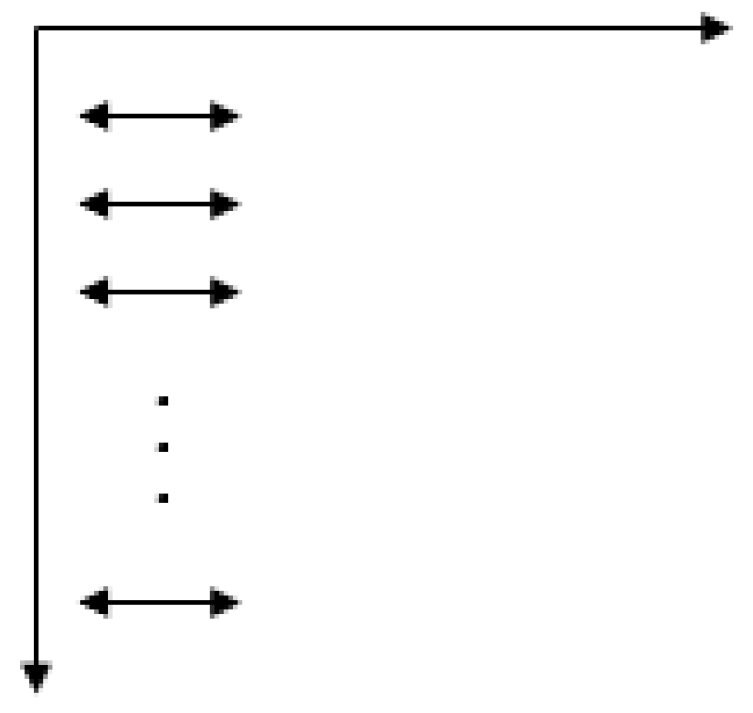
The horizontal traversal.

In the previous section, the number of blocks with the best classification effect has been obtained. Next, it is the main problem of this paper to quantify the texture relationship between blocks by means of mutual information. For the calculation of mutual information, we proposed three calculation modes, namely horizontal traversal, vertical traversal and eight-neighborhood traversal. Calculating mutual information of adjacent blocks by the horizontal traversal as shown in Figure 12.

According to Formula (5), the mutual information between adjacent blocks $x_{1}$ and $x_{2}$, $x_{2}$ and $x_{3}$,⋯, $x_{m\times n-1}$ and $x_{m\times n}$ is calculated by horizontal traversing from the first row to the last. They are $I_{1}{}^{r}$, $I_{2}{}^{r}$, ⋯, $I_{m\times \left(n-1\right)}{}^{r}$. In order to facilitate the next classification and recognition research, the m×(n−1) mutual information obtained above is stacked, and then a feature vector $R_{r}$ is obtained, as in Formula (7).

(7) $$R_{r}=\left(\begin{array}{c} \begin{array}{c} I_{1}^{r}\\ I_{2}^{r}\\ \vdots \end{array}\\ I_{m\times \left(n-1\right)}^{r} \end{array}\right)$$

**Figure 13:**
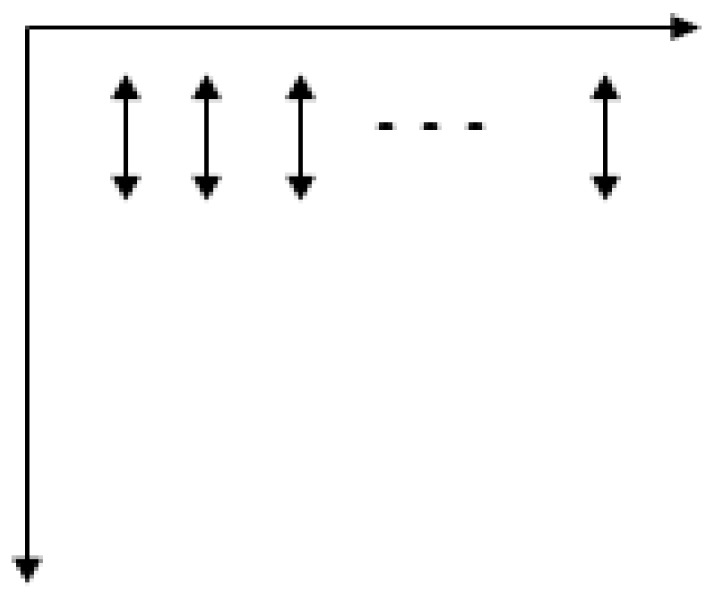
The vertical traversal.

The vertical traversal mode as shown in Figure 13.

Similarly, the mutual information between the adjacent blocks $x_{1}$ and $x_{n+1}$, $x_{n+1}$ and $x_{2n+1}$, ⋯ ,$x_{m\times \left(n-1\right)}$ and $x_{m\times n}$ is calculated by vertical traversal from the first column to the last. The n×(m−1) mutual information obtained above is stacked, and then a feature vector $R_{c}$ is obtained, as in Formula (8).

(8) $$R_{c}=\left(\begin{array}{c} \begin{array}{c} I_{1}^{c}\\ I_{2}^{c}\\ \vdots \end{array}\\ I_{n\times \left(m-1\right)}^{c} \end{array}\right)$$

**Figure 14:**
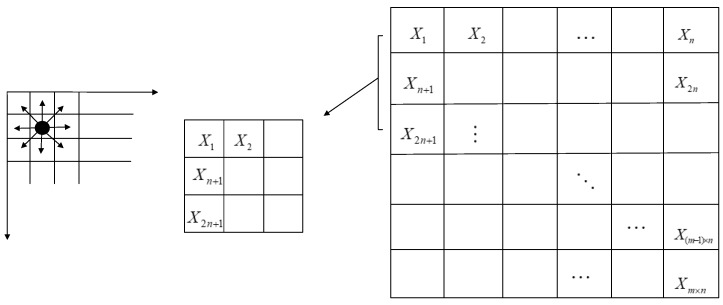
The eight-neighborhood traversal.

The eight-neighborhood traversal mode as shown in Figure 14.

First, calculate the mutual information of block $x_{n+2}$ and its surrounding eight neighbors $x_{1}$, $x_{2}$, $x_{3}$, $x_{n+1}$, $x_{n+3}$, $x_{2n+1}$, $x_{2n+2}$, $x_{2n+3}$, then calculate the eight neighborhood mutual information of $x_{n+3}$, $x_{n+4},\cdots$,$\;x_{\left(m-1\right)\times n-1}$, which are respectively $I_{1}{}^{e}$, $I_{2}{}^{e}$, ⋯, $I_{\left(m-2\right)\times \left(n-2\right)\times 8}{}^{e}$. Performing a stacking operation on (m−2)×(n−2)×8 mutual information to obtain a feature vector $R_{e}$, as in Equation (9).

(9) $$R_{e}=\left(\begin{array}{c} \begin{array}{c} I_{1}^{e}\\ I_{2}^{e}\\ \vdots \end{array}\\ I_{\;\left(m-2\right)\times \left(n-2\right)\times 8}^{e} \end{array}\right)$$

In this paper, the training set and test set are processed separately in the above three calculation modes, and then the optimal mutual information calculation mode is determined by the experimental results.

### 3.4. Classification Identification

The above mentioned that the bit plane is processed by 20 × 20 blocks, and then the mutual information between the blocks is calculated in three modes of horizontal, vertical and eight-neighborhood. Furthermore, the feature vectors $R_{r}$, $R_{c}$ and $R_{e}$ are obtained as the feature extraction of dorsal hand vein. The training samples $R_{t}^{\prime }$ from the device 1, and the test samples $R_{t}$ from the device 2. The feature vector of the training samples in the three calculation modes is defined as $R_{t}^{\prime }=\left[I_{t1}^{\prime }\;I_{t2}^{\prime }\;...\;I_{tk}^{\prime }\right],\;t=1,2,...,n$, and the feature vector of test samples is defined as $R_{t}^{\;}=\left[I_{t1}^{\;}\;I_{t2}^{\;}\;...\;I_{tk}^{\;}\right],\;t=1,2,...,n$, where t represents the category of samples, and k is the number of mutual information.

We have carried out experiments in the cross-device and single-device scenario. In the single-device scenario, there are 10 images of each hand, we only took five samples to match. In the cross-device scenario, we also took five samples in the test sample of device two to match. An n-dimension distance vector matrix $dis_{t}\left(t=1,2,3,4\cdots ,n\right)$ is obtained by calculating the Euclidean distance [20] between the test sample (from device 2) feature $R_{t}$ and training sample (from device 1) features $R_{t}{}^{\prime }$, as in Formula (10).

(10) $$dis_{t}=\left|\left|R_{t}-R_{t}{}^{\prime }\right|\right|=\sqrt{\sum_{i=1}^{k}{\left(I_{ti}-I_{ti}^{\prime }\right)}^{2}},t=1,2,\cdots ,n$$

Get the minimum value d of the feature distance.

(11) $$d=\underset{t=1}{\overset{n}{min}}\left\{dis_{t}\right\}$$

Then the test sample $R_{t}$ is identified as the training sample $R_{t}{}^{\prime }$ through the minimum value d.

## 4. Experiment Analysis

In order to fully prove the result of cross-device hand vein image recognition based on the bit-plane mutual information, this experiment used two different parameters of the device, labeled as first device and second device to collect and classify 50 peoples’ hand vein images. Their right and left hand were collected by 10 images, respectively; a total of 2000 dorsal hand vein images with a size of 400×400 were taken. Due to the disparity between the vein networks, right and left hands are considered as different subjects, which makes the number of classes double. In addition, there are differences in parameters such as contrast, brightness, focal length, and lens optical performance of two different devices. The data were collected twice by different devices with a time span of 12 months.

The experiment uses one device for registration and the other for recognition. Data acquisition uses two generations of different acquisition systems. The two devices are two generations of different acquisition systems, their illumination module adopts reflectance illumination scheme of infrared LED array with different wavelength and bandwidth. Device1 uses the 700 nm ~ 1000 nm near-infrared diode source (wideband source) as the active incident source. Device2 uses the near-infrared diode light source with a central band of 850 nm and a radius bandwidth of 50 nm (narrow-band light source) and increases the number of LED array. In the image acquisition module, device1 uses a common camera, the main parameters are as follows: Resolution: 420 lines, output pixels: 640×480, signal to noise ratio: 40 dB, device2 uses an industrial grade camera, the main parameters: Resolution: 570 lines, output pixels: 768×494, signal to noise ratio: 46 dB. In the interface module, the two devices also use different acquisition cards.

In order to ensure the distribution of cross-device dorsal hand vein images, it used automatic collection and didn’t limit the volunteers’ posture. In addition, the parameter difference between different devices makes it more difficult for recognition based heterogeneous images. We used different types of images (gray-normalized image, binary image, the gray image that only retains the contour of dorsal hand vein and the bit plane image) to experiment separately. This experiment chose optimal number of blocks, bit plane and mutual information calculation mode to compare the result of our algorithm with other algorithms for cross-device images, and then the robustness of the algorithm was verified by the recognition rate.

Due to changes of the collection environment, the images collected by two devices are significantly different, which are mainly reflect in the changes of brightness, displacement and rotation.

**Figure 15:**
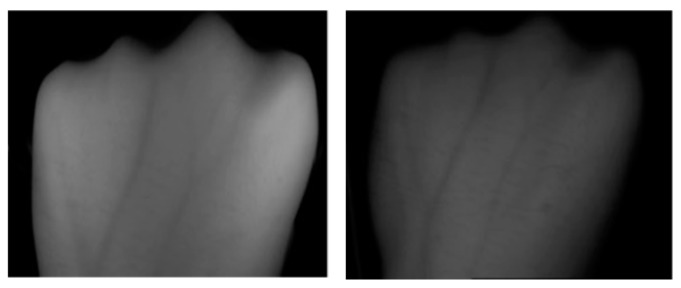
Difference in brightness.

The images have a distinct brightness difference in the brightest and darkest areas as shown in Figure 15. it affects the recognition rate to a large extent.

**Figure 16:**
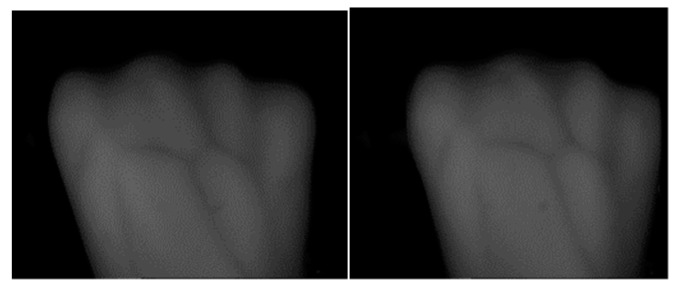
Difference in displacement.

The difference in the posture of the person and the handle width of different devices, the back of hand produces a certain displacement, as shown in Figure 16. When the displacement is large, some information on the back of hand will be covered, therefore, it affects the recognition rate to a certain extent.

**Figure 17:**
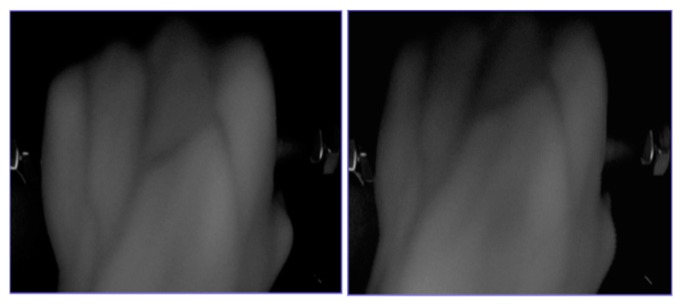
Difference in rotation angle.

Since the different angles of collector’s hands, dorsal hand vein images are deformed, as shown in Figure 17. It can also affect the recognition rate of dorsal hand vein.

These differences can lead to a significant increase in the difficulty and complexity of recognition of cross-device dorsal hand vein images. Experimental comparison is conducted below to verify that method of this paper has a better effect on overcoming the effects of brightness, displacement and rotation.

First, the gray-normalized image (Figure 4), the binary image (Figure 5) and the gray image that only retains the contour of dorsal hand vein (Figure 6) were divided into 20 × 20 blocks, respectively. Then, the mutual information feature vector between the blocks was obtained by using the calculation modes of horizontal, vertical and eight-neighborhood respectively. Finally, the classification result was output by the Euclidean distance classifier. The recognition rates of three different types of dorsal hand vein images in three modes are shown in Table 1.

Through experiments, it can be found that the recognition rate of the gray-normalized image is less than 50%, and the binary image reaches 86.60%, while the gray image that only retains the contour of dorsal hand vein reaches 89.67%. The gray-normalized image has the effect of the background such as skin, and the binary image completely loses the grayscale information, so the recognition rate is not as good as the gray image that only retains the contour of dorsal hand vein. In the three modes, the recognition rate of the eight-neighborhood mode is higher than the other two modes, which indicates that it is more accurate to calculate the mutual information of adjacent blocks by eight-neighborhood traversal as the texture feature of dorsal hand vein.

**Figure 18:**
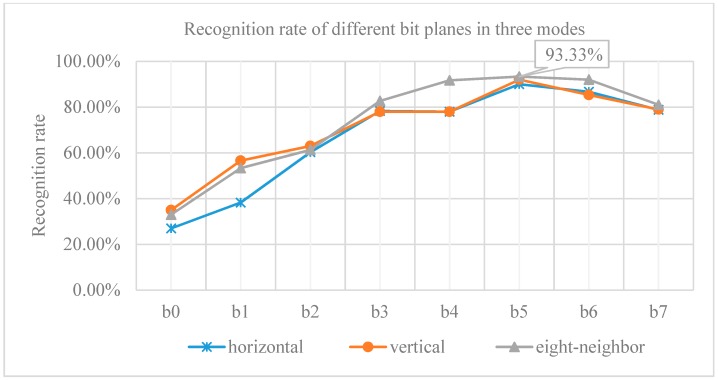
Recognition rate of different bit planes in three modes.

In order to make full use of the gray information of the dorsal hand vein and overcome the effects of illumination, brightness, rotation and scale changes in the acquisition environment, the eight bit planes generated by the gray image that only retains the contour of dorsal hand vein was tested separately, and the statistical recognition rate is shown in Figure 18.

It can be seen that when the number of blocks is 20 × 20 and the mutual information calculation mode is eight-neighborhood traversal, the recognition rate of the sixth bit plane (b5) reaches the best in this paper, which is 93.33%. The sixth bit plane not only contains the original contour of dorsal hand vein, but also overcomes the influence of brightness and noise to a certain extent, and better reflects the texture features. At the same time, the experiment is compared with other methods on dorsal hand vein recognition. In the long-term research of the dorsal hand vein recognition, the Intelligent Recognition and Image Processing Laboratory of North China University of Technology (NCUT) reproduced some mainstream algorithms on the NCUT hand vein dataset. The results of the comparative experiment are shown in Table 2.

The LBP algorithm is used to research the local grayscale texture features, and it requires a high degree of registration about the position of dorsal hand vein, so the recognition rate is not high. The PCA algorithm treats the sample as a whole, and therefore ignores the local attribute, but the neglected part is likely to contain important separability information, so the effect of cross-device dorsal hand vein recognition is very poor. Although the SIFT algorithm has the characteristics of scale transformation, rotation and illumination invariance, there are fewer feature points taken by different devices, therefore, the recognition rate is also not very high. The position of the feature points generated by the Gaussian random distribution based on the GDRKG random feature point algorithm is not determined, so the probability of matching errors is greatly increased, and the recognition rate is not ideal. The improved SIFT algorithm has achieved a good recognition rate in cross-device experiments, but it relies too much on parameter settings and template selection, and the calculation speed is very slow. Our method is to calculate the mutual information between adjacent blocks of the bit planes to quantify the texture features of dorsal hand vein, and the Euclidean distance is used for classification. The high recognition rate achieved by the experiment fully demonstrates the effectiveness and feasibility of the proposed method.

## 5. Conclusions

Aiming at the problem that the recognition rate of the dorsal hand vein image collected by different devices is not high, this paper proposes a research method-based bit plane and block mutual information. The optimal block is determined by the variance corresponding to the average entropy matrix, the gray-normalized image, the binary image, the gray image that only retains the contour of dorsal hand vein, and the bit planes are tested respectively under various mutual information calculation modes. By comparing other algorithms used on cross-device hand vein recognition, the method proposed in this paper has been significantly improved. However, at present, only the one-bit plane is processed separately, therefore, the fusion and optimization of multiple bit planes will be the focus of further research in the later stage.

## Figures and Tables

**Table 1:** Recognition rates of three different types of dorsal hand vein images in three modes.

Modes	Gray-Normalized	Binary	The Gray Image that Only Retains the Contour
Horizontal	48.30%	43.30%	46.33%
Vertical	86.60%	83.30%	83.30%
Eight-neighborhood	88.00%	86.70%	89.67%

**Table 2:** Comparison of recognition rates about different algorithms under cross-device.

Algorithms	Recognition Rate (Single-Device)	Recognition Rate (Cross-Device)
Ours	>98%	93.33%
LBP	93.50%	73.42%
PCA	90.40%	54.83%
SIFT	97.50%	86.60%
Gaussian distribution based random key-point generation (GDRKG)	92.30%	71.38%
Improved SIFT	98.63%	90.8%
